# Editorial for the Special Issue on Exploring IoT Sensors and Their Applications: Advancements, Challenges, and Opportunities in Smart Environments

**DOI:** 10.3390/mi15081048

**Published:** 2024-08-18

**Authors:** Lei Jing, Yoshinori Matsumoto, Zhan Zhang

**Affiliations:** 1 Graduate School of Computer Science and Engineering, The University of Aizu, Tsuruga, Ikki-machi, Aizuwakamatsu 965-8580, Japan; 2 Department of Applied Physics and Physico-Informatics, Keio University, Yokohama 223-8522, Japan; 3 School of Computer Science and Technology, Harbin Institute of Technology, Harbin 150001, China

As the editor of the Special Issue on “Exploring IoT Sensors and Their Applications: Advancements, Challenges, and Opportunities in Smart Environments”, I am delighted to present this collection of groundbreaking research that addresses the emerging needs and challenges in the field of IoT sensors and smart environments. This Special Issue brings together ten contributions that showcase the significant advancements and innovative applications of IoT sensors, providing valuable insights into the current state of the art and highlighting future research directions.

The field of IoT sensors has rapidly evolved, driven by the increasing demand for smart solutions in healthcare, industry, and everyday life. Recent studies, such as Deng et al. [1], have highlighted the growing importance of edge computing in reducing latency and improving the efficiency of IoT systems. Similarly, Li et al. [2] have explored the integration of wireless sensor networks (WSNs) with IoT, emphasizing the need for low-power, high-reliability communication protocols.

However, several challenges remain, including the need for more accurate, reliable, and energy-efficient sensors, as well as the integration of these sensors into comprehensive systems that can effectively process and analyze the vast amounts of data they generate. For instance, Deng et al. [3] underscore the ongoing efforts to develop power management strategies that extend the operational life of IoT devices, which is critical for applications in remote and resource-constrained environments.

This Special Issue addresses these gaps by presenting research that not only introduces new sensor technologies but also explores novel algorithms and systems that enhance the performance and utility of these sensors in various applications.

The following contributions in this Special Issue cover a broad range of applications and technologies, each addressing specific gaps in current knowledge:

Nguyen et al. [4] present the development of flexible pressure sensors designed to monitor human walking phases. The sensors are integrated with machine learning algorithms, specifically tailored to accurately capture and interpret the complex biomechanical processes involved in human gait. This work addresses the challenge of creating wearable devices that can provide real-time, reliable data for applications in healthcare and rehabilitation. By focusing on the integration of flexible materials and advanced algorithms, the authors have laid the groundwork for future innovations in wearable health monitoring systems. 

Vu et al. [5] propose a health monitoring system that leverages Pyralux copper-clad laminate film as the sensor material, combined with a random forest algorithm for data analysis. This system is designed to enhance the accuracy and reliability of health monitoring, particularly in environments where traditional sensors may be less effective. The paper highlights the potential of combining flexible sensor materials with robust machine learning techniques to create more effective health monitoring systems, paving the way for broader applications in remote health care and wearable devices.

Fan et al. [6] introduce a smart-data glove designed for gesture recognition in amphibious communication scenarios. The glove integrates multiple sensors to capture hand movements and gestures, which are then processed to enable effective communication in challenging environments, such as underwater. The authors address the gap in reliable communication methods for environments where traditional devices fail, demonstrating how wearable technology can be adapted for specialized use cases.

Li et al. [7] present HRBUST-LLPED, a benchmark dataset specifically designed for the development and testing of wearable low-light pedestrian detection systems. The dataset addresses the critical need for reliable pedestrian detection in low-visibility conditions, such as at night or in poorly lit environments. The authors provide a comprehensive analysis of the dataset and demonstrate its potential for improving pedestrian safety through enhanced detection algorithms.

In this paper, Javeed et al. [8], the authors explore the use of biosensors in multimodal deep human locomotion decoding, facilitated by the Internet of Healthcare Things (IoHT). This research focuses on integrating multiple biosensor data streams to create a comprehensive understanding of human locomotion patterns, which is crucial for applications in health monitoring, rehabilitation, and sports science. The study highlights the potential of IoHT to enhance the accuracy and utility of wearable health monitoring systems by leveraging deep learning techniques.

In research by Chae et al. [9], the authors introduce a low-power IGZO memristor-based gas sensor designed for detecting isopropanol alcohol gas, embedded within an IoT monitoring system. The authors address the challenge of energy consumption in sensor networks by developing a gas sensor that is both highly sensitive and energy-efficient. This work is particularly relevant for applications in industrial safety and environmental monitoring, where continuous, reliable detection of volatile compounds is essential.

In a paper by Tajitsu et al. [10], the authors present an innovative application of braided piezoelectric poly-l-lactic acid (PLLA) cord sensors in a sleep bruxism detection system. The authors focus on developing a sensor that minimizes physical and mental stress, which is crucial for ensuring user compliance and comfort during long-term health monitoring. The sensor’s application in detecting bruxism—a condition that can lead to severe dental issues if untreated—demonstrates the potential of wearable IoT sensors in providing non-invasive, stress-free health monitoring solutions.

Yin et al. [11] investigate the geometry scaling of microfluidic devices used for deterministic lateral displacement (DLD) to separate multi-size particles. The authors introduce an externally balanced cascade design that enhances the separation efficiency of microfluidic systems. This work addresses the challenge of precisely controlling particle separation in lab-on-a-chip devices, which has significant implications for biomedical research and diagnostics.

Sonchan et al. [12] investigate robust orientation estimation using MEMS MARG modules, which integrate magnetic, angular rate, and gravity sensors. The authors develop algorithms that improve the accuracy and reliability of orientation estimation, which is critical for human-computer interaction systems, including virtual reality (VR) and augmented reality (AR) applications. By enhancing the performance of MARG modules, this research contributes to more immersive and responsive user experiences in HCI systems.

Finally, Yu et al. [13] demonstrate the application of gesture-controlled robotic arms in agricultural harvesting, utilizing a data glove with bending sensors and OptiTrack systems, which underscores the growing importance of IoT sensors in precision agriculture. This innovative application of IoT sensors in agriculture addresses the challenge of improving precision and efficiency in harvesting operations. The integration of gesture recognition technology with robotic systems represents a significant step forward in the development of smart agricultural solutions.

While the advancements presented in this Special Issue represent significant progress, there remain several avenues for future research. Interdisciplinary collaboration will be crucial in further integrating IoT sensors with AI and data science to create smarter, more autonomous systems. Additionally, the scalability and miniaturization of sensors, coupled with advancements in battery life and energy harvesting, will be vital for the continued expansion of IoT applications. Moreover, ensuring the security and privacy of the vast amounts of data generated by IoT systems will require ongoing attention, particularly as these technologies become more embedded in our daily lives.

In conclusion, this Special Issue has provided a comprehensive overview of the current advancements in IoT sensors and their applications in smart environments. The contributions have addressed critical gaps in the field and set the stage for future innovations. I look forward to witnessing the continued evolution of this dynamic field, driven by the collaborative efforts of researchers and practitioners worldwide.
